# Clinical presentation of hemophagocytic lymphohistiocytosis in adults is less typical than in children

**DOI:** 10.6061/clinics/2016(04)05

**Published:** 2016-04

**Authors:** Zuojuan Zhang, Juandong Wang, Buqiang Ji, Tatiana von Bahr Greenwood, Yuan Zhang, Yongjing Wang, Dexiao Kong, Ai Li, Yang Jiang, Yanan Guo, Xiaoli Liu, Yingxue Wang, Aixia Dou, Nailin Li, Jan-Inge Henter, Guizhen Sun, Chengyun Zheng

**Affiliations:** I The Second Hospital of Shandong University, Department of Hematology, Jinan, China; II Shandong University, Institute of Biotherapy for Hematological Malignancies, Jinan, China; III Linyi People's Hospital, Department of Hematology, Linyi, China; IV Childhood Cancer Research Unit, Department of Women's and Children's Health, Karolinska Institute, Karolinska University Hospital, Stockholm, Sweden; V the Second Hospital of Shandong University, Center of Evidence-based Medicine, Jinan, China; VI Karolinska Institutet at Karolinska University Hospital (Solna), Department of Medicine-Solna, Clinical Pharmacology Unit, Stockholm, Sweden; VII Shandong University-Karolinska Institutet Collaborative Laboratory for Stem Cell Research, Jinan, China

**Keywords:** Hemophagocytic Lymphohistiocytosis, Clinical Characteristics, Adult, Pediatric

## Abstract

**OBJECTIVE:**

Hemophagocytic lymphohistiocytosis in adults is largely underdiagnosed. To improve the rate and accuracy of diagnosis in adults, the clinical and laboratory characteristics of hemophagocytic lymphohistiocytosis were analyzed in and compared between adults and children in a Chinese cohort.

**METHOD:**

Data from 50 hemophagocytic lymphohistiocytosis patients, including 34 adults and 16 children who fulfilled the 2004 hemophagocytic lymphohistiocytosis diagnostic criteria, were collected and analyzed.

**RESULTS:**

1. Etiological factors: The proportion of Epstein-Barr virus infection was lower in adults compared with children, whereas fungal infection and natural killer/T cell lymphoma were more frequent in adults (*P*<0.05). 2. Clinical manifestations and laboratory findings: Over 90% of adults and pediatric patients presented with fever, thrombocytopenia and high serum ferritin levels. However, in adults, the proportions of hepatomegaly, splenomegaly and jaundice were much lower (*P*<0.01) than in children, and serous cavity effusion was more frequent in adult patients (*P*<0.05). More children had hemoglobin <90 g/L, total bilirubin >19 mmol/L and lactate dehydrogenase >500 U/L compared with adults (*P*<0.05). 3. The time interval from the onset of symptoms to clinical diagnosis was significantly shorter in pediatric patients than in adults (*P*<0.05).

**CONCLUSIONS:**

Certain clinical features were different between the two groups. The less characteristic clinical presentation of hemophagocytic lymphohistiocytosis in adults may make the disease more difficult to diagnose. Our findings suggest that hemophagocytic lymphohistiocytosis should be considered when an adult patient presents with the above-mentioned symptoms.

## INTRODUCTION

Hemophagocytic lymphohistiocytosis (HLH) is a severe inflammatory disorder characterized by a significant accumulation of activated CD8^+^ T lymphocytes and histiocytes in the bone marrow (BM) and lymphoid tissues. The cytokine storm resulting from the accumulation of activated immune cells leads to fever, hepatosplenomegaly, impaired liver function and other clinical and laboratory manifestations of HLH 1,2-3. Classically, HLH is divided into primary or familial HLH (FHL) and secondary HLH (sHLH). FHL, which is an autosomal recessive disease, is caused by mutations in the genes encoding the molecules involved in the granule exocytosis machinery of cytotoxic T lymphocytes and natural killer (NK) cells. Although the pathogenesis of sHLH is largely unknown, the two forms of HLH, or sHLH and FHL, have similar clinical characteristics 1,2-3.

HLH is characterized by fever, hepatosplenomegaly, bicytopenia or pancytopenia, hemophagocytosis in the BM and lymphoid tissues, liver dysfunction and central nervous system (CNS) symptoms 1,2-3. Infection is described as a major trigger of HLH development, and Epstein-Barr virus (EBV) has been described as the dominant pathogen 1, 3. Without effective treatment, many HLH patients die within 3 months. Treatment of HLH, whether in the FHL or the sHLH form, has significantly improved due to early introduction of immunochemotherapy 2, 4. Obviously, an early diagnosis of HLH and a timely initiation of effective treatment are crucial for improving the prognosis of HLH patients. To this end, a good understanding of the clinical features of adult HLH would be very helpful to clinicians for prompt diagnosis and effective treatment. Notably, however, previous reports on HLH have mainly focused on pediatric HLH, and current knowledge of HLH primarily originated from observations of pediatric patients. Therefore, the clinical features of adult HLH have not been well described. The present retrospective clinical investigation was performed to characterize the clinical and laboratory features of adult HLH in a Chinese cohort and to compare adults and children with HLH in this cohort.

### Patients

A total of 86 patients diagnosed with HLH on admission or by discharge from January 2008 to December 2012 in the Departments of Hematology and Pediatrics of the Second Hospital of Shandong University and Linyi People's Hospital, Shandong, China, were studied. Fifty of the 86 patients met the 2004 HLH diagnostic criteria 2; these individuals constituted the cohort of the present study. A diagnosis of HLH was made when five or more of the following eight criteria were fulfilled: 1) fever; 2) splenomegaly; 3) cytopenia affecting at least two of the three lineages in the peripheral blood (hemoglobin < 90 g/L, platelets <100×10^9^/L, neutrophils <1.0×10^9^/L); 4) hypertriglyceridemia and/or hypofibrinogenemia (fasting triglycerides ≥3.0 mmol/L or ≥3 SDs, fibrinogen ≤1.5 g/L or ≤3 SDs); 5) ferritin ≥500 μg/L; 6) soluble CD25 (soluble IL-2 receptor) ≥2400 U/mL; 7) low or absent NK cell activity; and/or 8) hemophagocytosis in the BM, spleen or lymph nodes. Clinical, laboratory and imaging data as well as information on etiological factors, such as infections (including with EBV, cytomegalovirus (CMV) and bacteria) or underlying diseases, from the 50 HLH patients were collected and analyzed. Approval for this study was obtained from the Ethics Committee of Shandong University.

According to the cut-off point for age (15 years old), the patients were divided into an adult group and a pediatric group. Patients who received therapy with steroids and/or intravenous immunoglobulin (IvIG) were grouped into a “non-specific HLH protocols” group, and those who underwent the 2004 HLH treatment protocol were grouped into an “HLH-2004 protocol” group.

### Laboratory Tests

#### EBV serology test and EBV-DNA quantification

EBV infection was determined by detecting serum anti-viral capsid antigen (VCA)-IgG, anti-VCA-IgM, and anti-early antigen (EA)-IgG using an anti-EBV antibody kit (EUROIMMUN Medizinische Labordiagnostika AG, Lubeck, Germany) following the procedures detailed by the manufacturer. EBV-DNA was detected using the quantitative real-time polymerase chain reaction (qRT-PCR) technique with a LightCycler 480/96 (fluorescence-based quantitative thermal cycler, Roche, Indianapolis, IN, USA) and EBV nucleic acid amplification fluorescence detection kits (KAIJIE Biological Engineering Co. Ltd., Shenzhen, China) following the manufacturers' protocols.

#### CMV serology test and CMV-DNA quantification

An enzyme-linked immunosorbent assay (ELISA) was applied to detect anti-CMV-IgM antibody and anti-CMV-IgG antibody in serum using anti-CMV antibody kits provided by EUROIMMUN AG (Lubeck, Germany) and a BEP-2000 automatic microplate reader (Dade, Miami, FL, USA) following the protocols provided by the manufacturers. CMV-DNA in serum was quantified by the qRT-PCR method using a CMV PCR Fluorescence Diagnosis Kit (Daan, Guangzhou, China) and a LightCycler 480/96 fluorescence-based quantitative thermal cycler (Roche, Indianapolis, IN, USA)

#### Detection of pathogenic bacteria and fungi

Blood samples from the patients were cultivated for detection of pathogenic bacteria and fungi using a BacT/ALERT 3D automated blood culture system (bioMérieux Inc., Durham, NC, USA) and a VITEK 2 compact microbial identification and susceptibility analysis system (bioMérieux S.A, Marcy l'Etoile, France).

#### Hemophagocytosis identification

Smears of BM were stained with Wright-Giemsa, and identification of hemophagocytosis was performed by hematopathologists. The histiocytes showing phagocytosis of platelets, one or more intact erythrocytes or precursors, neutrophils or granulocyte precursors, plasma cells, and/or lymphocytes were counted as indicating hemophagocytosis.

#### Routine blood tests

Blood routine tests were performed using a Sysmex XT-4000i Automated Hematology Analyzer (Mundelein, IL, USA) and kits from Beckman Coulter Trading Co. Ltd. (Shanghai, China).

#### Ferritin quantification

Ferritin in serum was quantified using a ferritin test kit purchased from Roche Diagnostics Corporation (Roche, Shanghai, China) and a Cobas e 601 analyzer in conjunction with ECL (Roche, Basel, Switzerland).

#### Other biochemistry tests

Quantification of serum lactate dehydrogenase (LDH) and total bilirubin (TB) were performed using a Beckman UniCel® DxC 800 automatic biochemistry analyzer (Brea, CA, USA) along with detection kits for LDH produced by Beckman Coulter Systems Ltd. (Suzhou, China). TB in serum was quantified by using a kit produced by Beijing Leadman Biochemical Technology Co. Ltd. (Beijing, China). Serum triglycerides, alanine aminotransferase (ALT), aspartate aminotransferase (AST) and gamma glutamyl transferase (γ-GT) were quantified using a Cobas C501 automatic biochemical analyzer (Roche Diagnostics, Mannheim, Germany) and respective related detection kits (Mannheim, Germany). Fibrinogen was measured using an STA Compact STAGO coagulation analyzer along with a kit manufactured by DIAGNOSTICA STAGO (Rue des Freres Chausson, Asnieres Cedex, France).

Splenomegaly, hepatomegaly, and lymphadenopathy in the abdomen were evaluated by color Doppler ultrasonography. Lymphadenopathy in the chest was determined by computed tomography (CT).

The early response to treatment was evaluated by comparing hemoglobin, platelets, neutrophils, ferritin, LDH and TB at diagnosis with the values after 2-3 weeks of HLH-2004 or non-specific HLH protocol treatment.

#### Statistical Analysis

The variables were described as follows: n, proportion and median. The categorical variables were compared using the χ^2^ test, and numeric variables were compared using the Wilcoxon signed-rank test. All analyses were performed using PASW Statistics 18.0, and a *P* value less than 0.05 was considered statistically significant.

## RESULTS

### Characteristics of HLH patients

The HLH patients included 34 adults and 16 children. There was no difference in the sex distribution (*P*>0.05). Etiological factors were different between the adult group and the pediatric group (*P*<0.05). In particular, 2.9% of the adults (1/34) had EBV infection, which was much lower than the rate in the children (31.3%, or 5/16). However, the proportions of fungal infection and NK/T cell lymphoma were higher in adults compared with children (*P*<0.05).

There was neither parental consanguinity nor a family history of HLH-like disease in any case. No genetic data were available for any of the HLH cases.

A total of 19 patients were treated with the HLH-2004 protocol, and 31 patients were administered non-specific HLH protocol treatment. None received stem cell transplantation (SCT). In all, 46% of patients (23/50) stopped treatment on their own, and 6 patients died (4 adults and 2 children) within 2 weeks of treatment initiation. In the remaining 21 cases, 12 individuals (7 adults and 5 children) were treated with the HLH-2004 protocol, and 9 (8 adults and 1 child) were treated with the non-specific HLH protocols. The results are shown in Table 1.

### Clinical manifestations and laboratory findings in HLH patients before treatment

The proportions of hepatomegaly, splenomegaly and jaundice were much lower in adults than in children (47.1% vs 93.8%, 61.8% vs 100.0%, and 41.2% vs 87.5%, respectively, *P*<0.01). Meanwhile, serous cavity effusion was present in 41.2% of adult patients, which was higher than the rate (12.5%) in children (*P*<0.05).

In total, 38.2% of adults had hemoglobin <90 g/L (*P*<0.05), compared with 75.0% of children. Additionally, more children than adults had TB >19 mmol/L and LDH >500 U/L (87.5% vs 41.2% and 93.8% vs 58.8%, respectively, *P*<0.05).

There were no differences in other clinical presentations and laboratory findings between the groups (*P*>0.05). The results are shown in Table 2.

### Time interval from onset to diagnosis and rate of early response to treatment

Our results demonstrated that the time interval from the onset of symptoms to clinical diagnosis was significantly shorter in pediatric patients than in adult patients (7 days vs 11 days, *P*<0.05).

Among the remaining 21 patients, 12 (7 adults and 5 children) were treated with the HLH-2004 protocol, and 9 (8 adults and 1 child) were treated with the non-specific HLH protocols. As shown in Table 3, after 2-3 weeks of treatment with the HLH-2004 protocol, the blood cells of the 12 patients (both adults and children) had recovered to different degrees. At the same time, serum ferritin and LDH levels had declined significantly in children (*P*<0.05). Interestingly, the non-specific HLH treatments also significantly reduced serum ferritin concentrations in adult HLH patients (data not shown).

## DISCUSSION

It has been previously reported that the onset age of FHL is usually less than 2 years old 1,2-3, whereas sHLH can occur at any age. In line with the earlier reports, our results showed that the age of pediatric patients at onset ranged from 2 months to 3 years, with a median age of 1 year. Our current knowledge of HLH has been largely obtained from studies of pediatric HLH, and particularly FHL. As a result, the clinical and laboratory features of adult HLH have not been fully characterized and were only recently described more extensively in larger reviews and studies. The present study showed that adult and pediatric HLH patients share certain clinical and laboratory features and triggering factors, although important differences do exist. This study specifically compared clinical manifestations and laboratory features of HLH in adults with those in pediatric patients.

Infection is a major trigger of HLH, and in children, EBV is the most common reason for HLH 1, 2, 5. As expected, we showed that EBV was the main infectious trigger of HLH in our pediatric patients, with a proportion of 31.3%. Surprisingly, we found that only 2.9% of the adult patients had HLH associated with EBV infection (*P*<0.05). In contrast, fungal infection and NK/T cell lymphoma were more common triggers in adults compared with children (*P*<0.05). These findings suggest a significant difference in triggering factors between adult and pediatric HLH in our specific cohort of patient. Notably, these results are not in line with the large recent review by Ramos-Casals in 2014 in which EBV infection was reported to be a common trigger of HLH in adults (6). However, 64.7% of the adults and 56.2% of the children had HLH with no identified etiological cause, so we needed to expend extra effort to determine the possible causes of sHLH in these groups.

It is well known that during the progression of HLH, activated CD8^+^ T cells produce large amounts of interferon-gamma (IFN-γ), which in turn stimulates overactivation and expansion of CD8^+^ T cells and macrophages. The activated T cells and macrophages infiltrate and accumulate in the liver, spleen, BM, lymph nodes and other organs or tissues, resulting in hypercytokinemia and tissue/organ damage, which in turn result in corresponding clinical manifestations 1,2,5,6. Typically, HLH patients present with long-term fever, hepatosplenomegaly, cytopenia, liver damage, jaundice, hemophagocytosis and CNS involvement 8. In our study, we found that adults and children with HLH shared the clinical manifestations of fever, thrombocytopenia, liver dysfunction, and elevated ferritin levels. However, adults with HLH less commonly presented with splenomegaly, hepatomegaly and jaundice compared with pediatric HLH patients. Interestingly, serous cavity effusion was found to have a much higher incidence in adults (41.2%) than in children (12.5%).

The differences observed in the degree of jaundice and in the incidence of hepatomegaly and splenomegaly between pediatric and adult patients may have been due to different conditions triggering the onset of HLH.

Cytopenia is an important laboratory manifestation observed in HLH, resulting from hemopoiesis suppression by the highly elevated levels of inflammatory cytokines, such as IFN-γ released by activated T cells, and from the phagocytosis of blood cells by over-activated macrophages. In our study, thrombocytopenia was common in both adults (97.1%) and children (93.8%), highlighting that thrombocytopenia is a prominent clinical feature of not only pediatric 8,9 but also adult HLH. However, cytopenia of multiple lineages was less common in adults than in children.

Ferritin presents in every cell type and serves as a carrier protein to store iron in a non-toxic form. An elevation of the serum ferritin concentration is linked to other conditions, such as infection, autoimmune and autoinflammatory disorders, and cancer, in addition to HLH. Excessively activated macrophages over-secrete ferritin, leading to the elevated serum ferritin levels observed in HLH. Elevated serum ferritin is an important marker not only for the diagnosis of HLH but also for the evaluation of disease activity 7,9,11. Decreased serum ferritin and LDH levels have been shown to reflect a good response to HLH treatments 8,12. Similar to ferritin, LDH (a cytoplasmic enzyme present in many cell types) is released upon cell damage, and LDH levels have also been suggested as a prognostic indicator of HLH 9. In the present study, we observed elevated levels of serum ferritin in nearly all patients and increased LDH levels in 58.8% of the adult and 93.8% of the pediatric patients. In line with previous studies, our findings showed that ferritin and/or LDH levels were significantly decreased after 2-3 weeks of treatment in both groups, and especially in pediatric patients, suggesting that serum ferritin and/or LDH may function as sensitive markers reflecting the early treatment response. It should be noted that the platelet count may also be a parameter reflecting the early response to disease treatment, given that in the present study, depressed platelet counts in pediatric HLH patients had increased after the first 2-3 weeks of treatment with both the HLH-2004 protocol and non-specific HLH treatment.

CNS involvement is considered to be an independent prognostic marker for pediatric HLH 13,15. Therefore, it is important to determine the status of CNS involvement in HLH patients at diagnosis. Previous studies demonstrated that 46% and 47% of pediatric HLH patients in Korean and Chinese cohorts, respectively, presented with CNS involvement 14,16. In our cohort, 29.4% and 37.5% of adult and pediatric HLH patients, respectively, showed CNS signs and symptoms at diagnosis. The value of CNS involvement in predicting prognosis in adult HLH patients needs to be further evaluated in future prospective studies with larger cohorts.

There are limitations to the present study. For example, we were unable to distinguish FHL from sHLH among the investigated pediatric and adult patients because no genetic data were available. Furthermore, the number of pediatric HLH patients in this study was relatively small. However, the incidences of the principal clinical and laboratory features in our pediatric patients were consistent with those in previous reports 1,2,5,7,17. Nevertheless, the retrospective nature of this study and the cohort size limited the possibility of prognostic analysis of clinical and laboratory parameters.

Altogether, the findings in this study demonstrate that consistent with pediatric HLH, fever, thrombocytopenia, liver dysfunction and elevated ferritin levels are common clinical manifestations in adult HLH. In contrast, splenomegaly, hepatomegaly and jaundice are less common and less severe in adults than in children, suggesting that this phenotype is not as common in adults with HLH. The time interval from onset to the clinical diagnosis of HLH in adults is significantly longer than in children, which may partly be due to less typical HLH manifestations in adult patients. Our investigation strongly suggests that the diagnosis of HLH needs to be kept in mind when encountering an adult patient presenting with fever, thrombocytopenia, an elevated serum ferritin level, and liver dysfunction.

## AUTHOR CONTRIBUTIONS

Zheng C, Sun G and Henter JI designed the study and revised the manuscript. Zhang Z, Wang J and Ji B collected and analyzed the data and wrote the manuscript. Zhang Y carried out the statistical analysis of the data and built the tables. Wang Y, Kong D, Li A, Jiang Y, Guo Y, Liu X,Wang Y and Dou A collected the data. Li N and von Bahr Greenwood T revised the manuscript.

## Figures and Tables

**Table 1:** Characteristics of hemophagocytic lymphohistiocytosis patients (n, %).

	Adult group (n=34)	Pediatric group (n=16)	*χ^2^*	*P*
Sex: Male	20 (58.8)	8 (50.0)	0.344	0.558
Female	14 (41.2)	8 (50.0)		
Age (years; median, range)	43 (15-77)	1 (0.2-3)	/	**0.000**
Etiological factors				
Bacterial infection	6 (17.6)	2 (12.5)	9.925	**0.042**
Fungal infection	2 (5.9)	0 (0.0)		
EBV infection	1 (2.9)	5 (31.3)		
NK/T cell lymphoma	3 (8.8)	0 (0.0)		
Unknown cause	22 (64.7)	9 (56.2)		
Treatment				
HLH protocol*	11 (32.4)	8 (50.0)	5.737	0.125
Non-HLH protocol				
Steroids	15 (44.1)	3 (18.8)		
Steroids and immunoglobulin	5 (14.7)	5 (31.2)		
Steroids and cyclosporine	3 (8.8)	0 (0.0)		
2-3 weeks of follow-up				
Abandonment of treatment	15 (44.1)	8 (50.0)	0.200	0.905
Death	4 (11.8)	2 (12.5)		
Partial or complete response	15 (44.1)	6 (37.5)		

**Table 2:** Clinical and laboratory findings in adult and pediatric HLH patients at diagnosis (n, %).

	Adult group (n=34)	Pediatric group (n=16)	*χ^2^*	*P*
Clinical presentations				
Fever	34 (100.0)	16 (100.0)	0.000	1.000
Hepatomegaly	16 (47.1)	15 (93.8)	10.068	0.002</emph>
Splenomegaly	21 (61.8)	16 (100.0)	8.267	**0.004**
Lymphadenopathy	9 (26.5)	7 (43.8)	1.493	0.222
Pulmonary involvement	23 (67.6)	8 (50.0)	1.438	0.230
Serous cavity effusion	14 (41.2)	2 (12.5)	4.112	**0.043**
Neurological symptoms	10 (29.4)	6 (37.5)	0.327	0.567
Hemorrhages	12 (35.3)	6 (37.5)	0.023	0.880
Jaundice	14 (41.2)	14 (87.5)	9.475	**0.002**
Urinary symptoms	5 (14.7)	0 (0.0)	2.614	0.106
Blood cell analysis				
Hemoglobin <90 g/L	13 (38.2)	12 (75.0)	5.882	0.015
Platelets <100×10^9^/L	33 (97.1)	15 (93.8)	0.310	0.578
Neutrophils <1×10^9^/L	22 (64.7)	12 (75.0)	0.530	0.467
Hemophagocytosis in BM	30 (88.2)	13 (81.3)	0.441	0.507
Blood biochemistry				
Ferritin >500 μg/L	33 (97.1)	16 (100.0)	0.480	0.488
Fibrinogen ≤1.5 g/L	16 (66.7)	7 (43.8)	0.048	0.827
ALT >40 U/L	28 (82.4)	15 (93.8)	1.174	0.279
AST >40 U/L	30 (88.2)	16 (100.0)	2.046	0.153
γ-GT >50 U/L	26 (76.5)	13 (81.2)	0.145	0.704
TB >19 mmol/L	14 (41.2)	14 (87.5)	9.475	0.002
LDH >500 U/L	20 (58.8)	15 (93.8)	6.320	0.012
TG >3.0 mmol/L	15 (44.1)	7 (43.8)	0.001	0.981
Na <135 mmol/L	18 (52.9)	8 (50.0)	0.038	0.846
Ca <2 mmol/L	18 (52.9)	6 (37.5)	1.039	0.308
CRP >10 mg/L	15 (44.1)	11 (68.8)	2.645	0.104

BM: bone marrow; ALT: alanine aminotransferase; AST: aspartate aminotransferase; γ-GT: gamma glutamyl transpeptidase; TB: total bilirubin; LDH: lactate dehydrogenase; TG: triglycerides; CRP: C-reactive protein

**Table 3:** Changes in laboratory findings between diagnosis and after 2-3 weeks of treatment with the HLH-2004 protocol in adult and pediatric patients (medians).

	Adult group (n=7)			Pediatric group (n=5)		
		**Pre-treatment**	**Post-treatment**	*P*	**Pre-treatment**	**Post-treatment**	*P*
Hemoglobin (g/L)	90.00	103.00	0.620	82.00	87.00	0.841
Platelet (×10^9^/L)	42.00	70.00	0.165	51.00	113.00	0.008
Neutrophil (×10^9^/L)	0.85	3.54	0.456	0.63	1.37	0.056
Ferritin (μg/L)	2000.00	407.10	0.053	1650.00	121.50	0.032
LDH (U/L)	1013.90	419.00	0.126	1511.00	299.50	0.008
TB (mmol/L)	17.65	12.83	0.383	110.50	16.67	0.056
